# Mesenchymal Stem/Stromal Cells: A Review for Its Use After Allogeneic Hematopoietic Stem Cell Transplantation

**DOI:** 10.3390/biom16010147

**Published:** 2026-01-14

**Authors:** Ali Durdu, Ugur Hatipoglu, Hakan Eminoglu, Turgay Ulas, Mehmet Sinan Dal, Fevzi Altuntas

**Affiliations:** Department of Hematology & Apheresis Unit, Ankara Oncology Training and Research Hospital, University of Health Sciences, 06200 Ankara, Türkiye; dr.alidurdu@gmail.com (A.D.); ugurugur2192@gmail.com (U.H.); hakaneminoglu13@gmail.com (H.E.); turgayulas@gmail.com (T.U.); faltuntas@gmail.com (F.A.)

**Keywords:** mesenchymal stem/stromal cells, allogeneic hematopoietic transplantation, graft-versus-host disease, poor graft function, extracellular vesicles, immune modulation

## Abstract

Mesenchymal stem/stromal cells (MSCs) exhibit broad differentiation capability and strong immunoregulatory potential mediated through intercellular communication and the release of diverse paracrine mediators. They represent a promising but still investigational therapeutic approach for managing complications associated with allogeneic hematopoietic stem cell transplantation (allo-HSCT). This review provides an updated synthesis of MSC biology, their bidirectional interaction with immune cells, and their functional contribution to the hematopoietic niche. It also evaluates current clinical evidence regarding the therapeutic roles of MSCs and MSC-derived extracellular vesicles (EVs) in acute and chronic graft-versus-host disease (aGVHD/cGVHD), as well as in poor graft function. Mechanistic insights encompass macrophage polarization toward an anti-inflammatory phenotype, inhibition of dendritic cell maturation, enhancement of regulatory T-cell expansion, and modulation of cytokine signaling pathways. Within the bone marrow milieu, MSCs contribute to stromal restoration and angiogenic repair. Recent phase II/III trials in steroid-refractory (SR)-aGVHD have demonstrated overall response rates ranging from 48 to 71%. Efficacy appears particularly enhanced in pediatric patients and with early MSC administration. Across studies, MSC therapy shows a favorable safety profile; however, heterogeneity in response and inconsistent survival outcomes remain notable limitations. For poor graft function, limited prospective studies indicate hematopoietic recovery following third-party MSC infusions, and combination approaches such as co-administration with thrombopoietin receptor agonists are under investigation. MSC-derived EVs emulate many immunomodulatory effects of their parental cells with a potentially safer profile, though clinical validation remains in its infancy. MSC-oriented interventions hold substantial biological and therapeutic promise, offering a favorable safety margin; however, clinical translation is hindered by product variability, suboptimal engraftment and persistence, and inconsistent efficacy across studies. Future directions should emphasize standardized manufacturing and potency assays, biomarker-driven patient and timing selection, optimized conditioning and dosing strategies, and the systematic appraisal of EV-based or genetically modified MSC products through controlled trials.

## 1. Introduction

MSCs were originally identified in bone marrow as fibroblast-like progenitors with self-renewal and multilineage differentiation potential [1]. Subsequent work established their trophic and immunomodulatory functions, positioning MSCs as key regulators of tissue homeostasis and repair [2,3,4,5,6,7]. In line with ISCT recommendations, the term “mesenchymal stromal cell” is preferred, as most clinical MSC preparations do not fulfil classical stem cell criteria at the single-cell level and are better defined by their paracrine and immunoregulatory activity [8].

MSCs orchestrate innate and adaptive immunity through direct contact and secretion of bioactive mediators, and they constitute a fundamental component of the hematopoietic stem cell (HSC) niche, supporting HSC survival, quiescence and renewal [9,10]. These properties form the biological rationale for their therapeutic deployment in transplantation medicine—particularly for SR-GVHD and graft failure (GF) following allo-HSCT [11,12]. However, clinical efficacy remains limited by poor in vivo persistence, inadequate homing and loss of paracrine function, underscoring the need for optimized manufacturing and delivery strategies [13].

MSC-derived extracellular vesicles (MSC-EVs), including exosomes (30–120 nm) and microvesicles (100–1000 nm) and apoptotic bodies (500–2000 nm), recapitulate many immunomodulatory effects of parental cells [9] by promoting peripheral tolerance through PD-L1/PD-L2 signaling [14]. Early clinical studies and ongoing trials suggest MSC-EVs may offer a cell-free alternative with improved safety, stability and standardized production [15].

This review synthesizes current mechanistic insights and clinical evidence on MSCs and MSC-EVs in the post-transplant setting, with emphasis on immune regulation, bone marrow microenvironmental repair, and treatment of acute GVHD and graft dysfunction.

## 2. Methods

A structured search of PubMed, Embase and ClinicalTrials.gov (January 2004–November 2025) was performed using the terms ‘mesenchymal stem cells’, mesenchymal stromal cells’, ‘extracellular vesicles’, ‘acute GVHD’, ‘chronic GVHD’, and ‘poor graft function’. Studies involving human subjects with extractable data on MSC dose, route, OR and survival were included. Case reports, preclinical studies, and incomplete datasets were excluded. Data abstraction was performed independently by three reviewers.

## 3. Mesenchymal Stem/Stromal Cells: Biology and Immunological Functions

### 3.1. Origin, Biology and Functions

MSCs were first described in the 1970s as fibroblast-like adherent cells isolated from bone marrow that formed colony-forming units with osteogenic potential. Subsequent studies demonstrated their capacity to differentiate into osteogenic, chondrogenic, and adipogenic lineages, establishing MSCs as a multipotent stromal progenitor population capable of supporting hematopoietic growth. By 1991, the term mesenchymal stem cell was introduced to define this self-renewing mesodermal cell type, distinct from fibroblasts despite their similar morphology in vitro [16,17].

The International Society for Cellular Therapy (ISCT) subsequently defined the standard immunophenotype of MSCs as positive for CD73, CD90, and CD105, and negative for CD14, CD34, CD45, CD11b, CD79, and HLA-DR. These criteria remain widely adopted for the characterization of MSCs derived from bone marrow, adipose tissue, umbilical cord, and other sources [18].

MSCs play critical roles in tissue repair and regeneration through their immunosuppressive, anti-inflammatory, and cytoprotective activities. They exert paracrine effects via secretion of vascular endothelial growth factor (VEGF), hepatocyte growth factor (HGF), transforming growth factor-β (TGF-β), and interleukin-10 (IL-10), as well as through the release of extracellular vesicles that modulate immune and stromal cell behavior [19,20]. Their lineage specification is governed by transcriptional regulators such as RUNX2 (osteogenic), SOX9 (chondrogenic), and PPARγ (adipogenic), which integrate local microenvironmental and mechanical cues to direct differentiation programs [20].

Under basal conditions, MSCs display low immunogenicity due to minimal expression of HLA class II molecules; however, exposure to inflammatory cytokines such as interferon-γ (IFN-γ) can induce HLA-DR expression. Despite this inducibility, MSCs are generally regarded as hypoimmunogenic and have been successfully used in allogeneic transplantation settings with limited immune rejection [9,18].

The advent of single-cell RNA sequencing (scRNA-seq) has enabled precise identification of stromal subpopulations in humans, consistent with lineage-tracing evidence showing a shared MSC surface phenotype; however, comparative scRNA-seq analyses reveal additional nomenclatural heterogeneity, even within the same species [21,22].

### 3.2. Interactions with Immune System Cells

Recent studies confirm that MSCs orchestrate both innate and adaptive immunity through direct cell–cell interactions and a complex secretome that influences T cells, B cells, macrophages, dendritic cells (DCs), neutrophils, and natural killer (NK) cells [23,24,25]. Macrophages are central targets of MSC regulation; by secreting PGE_2_, TGF-β and CCL2, MSCs induce a shift from pro-inflammatory M1 toward anti-inflammatory M2 phenotypes, thereby supporting tissue tolerance [26,27]. Rather than producing IL-17 themselves, MSCs suppress Th17-driven inflammation and consequently limit IL-17–mediated neutrophil activation. Bone-marrow-derived MSCs decrease reactive oxygen species (ROS) release from activated neutrophils, whereas umbilical-cord-derived MSCs attenuate neutrophil-associated inflammation [28].

Mechanistically, MSCs and their extracellular vesicles restrain dendritic cell maturation by downregulating MHC-II, CD80, and CD86 expression and promoting an IL-10-high, IL-12-low tolerogenic phenotype [29]. By maintaining macrophages and DCs in an immature or regulatory state, MSCs attenuate effector T-cell activation and promote expansion of regulatory T cells (Tregs) [30,31]. This Treg induction is driven by soluble factors such as PGE_2_, TGF-β_1_, IL-10, HLA-G5, and indoleamine-2,3-dioxygenase (IDO), which collectively up-regulate FOXP3 and suppress alloreactive responses. IDO catabolizes tryptophan into kynurenine, inducing T-cell anergy and driving metabolic reprogramming of monocytes and DCs toward a tolerogenic phenotype [32,33,34,35]. As demonstrated by Kadri et al. IFN-γ-activated MSCs enhance IDO and HLA-G expression, promoting metabolic polarization of myeloid cells and expansion of suppressive Tregs through the kynurenine-AHR axis, thereby amplifying their immunoregulatory potency [36].

In addition to cytokines and enzymes, MSCs secrete galectin-1 and galectin-9, which modulate cytokine profiles and suppress allo-reactive CD4^+^ and CD8^+^ T-cell responses in GVHD models [37,38]. MSCs also release extracellular vesicles (EVs) that carry miRNAs and immunomodulatory proteins reflecting their parental cells’ function. Draguet et al. demonstrated that MSC-derived EVs are enriched in miR-21, miR-146a, miR-155, and PD-L1, which repress Th17 polarization and enhance Treg activity via TGF-β/Smad and PD-1/PD-L1 signaling pathways, providing a cell-free extension of MSC-mediated immunomodulation [39].

Finally, MSCs express Toll-like receptors (TLR-3 and TLR-4), and their ligation activates NF-κB, increasing CXCL10, IL-6, and IL-8 production. This pro-inflammatory shift temporarily reduces MSCs immunosuppressive capacity during infection, highlighting the context-dependent plasticity of MSCs immunoregulation [40].

### 3.3. Tissue Protection and Regeneration

Beyond their immunomodulatory effects, MSCs protect and repair damaged tissues through paracrine signaling that activates endogenous regenerative pathways in recipient cells [41]. In hematology, MSCs are recognized as key regulators of bone marrow stromal integrity and potential therapeutic agents for poor graft function (PGF) following allogeneic hematopoietic stem cell transplantation. Through secretion of stem cell factor (SCF), CXCL12, IL-6, and vascular endothelial growth factor (VEGF), MSCs support hematopoietic stem and progenitor cell (HSPC) survival, homing, and proliferation within the marrow niche [12].

In the setting of PGF, where the stromal niche is disrupted or functionally depleted, exogenously infused MSCs restore stromal integrity and reconstitute the hematopoietic microenvironment. MSCs also release angiogenic mediators including VEGF, hepatocyte growth factor (HGF), and angiopoietin-1, which promote endothelial repair, microvascular regeneration, and improved marrow perfusion [42]. Their immunomodulatory properties—marked by suppression of alloreactive T and natural killer cells, expansion of regulatory T cells, and rebalancing of cytokine profiles toward an anti-inflammatory state—further protect the marrow from immune-mediated injury [42].

These paracrine and extracellular vesicle-mediated effects confer anti-apoptotic and anti-fibrotic benefits through factors such as HGF, TGF-β, and matrix metalloproteinases (MMP-2, MMP-9), which enhance progenitor cell survival and remodel the extracellular matrix [43]. Collectively, these mechanisms provide a strong biological rationale for MSC-based therapy in PGF, although clinical efficacy remains variable depending on cell source, dose, and timing of infusion [44].

### 3.4. Beyond the Cells: Exosomes (MSC-EVs)

Initially thought to represent cellular debris from membrane degradation, exosomes are now recognized as key mediators of intercellular communication [45]. These nanosized extracellular vesicles originate from the endosomal multivesicular body pathway and are released through exocytosis. The immunological activity of exosomes was first demonstrated in 1996 by Raposo et al., who showed that B cell–derived exosomes bearing MHC class II molecules can present antigens to CD4^+^ T cells, thereby shaping adaptive immune responses [46].

Exosomes carry a complex molecular cargo—including proteins, lipids, mRNAs, microRNAs, and long non-coding RNAs—that enables targeted modulation of recipient-cell gene expression and signaling [46,47]. They are distinct from larger microvesicles (also called “macrovesicles,” 100–1000 nm), which are generated by outward budding of the plasma membrane rather than endosomal pathways. Consequently, exosomes typically contain endosome-associated proteins (Alix, TSG101, CD63, CD81), whereas microvesicles are enriched in cytoskeletal components, phosphatidylserine, and membrane-anchored receptors reflective of the parent cell surface [48].

Mesenchymal stromal/stem cells (MSCs) exert part of their immunomodulatory function through the release of extracellular vesicles (MSC-EVs) within their secretome—a mixture of cytokines, chemokines, and growth factors that regulate immune cell activation [9]. MSC-derived exosomes suppress Th17 differentiation and decrease secretion of pro-inflammatory cytokines such as TNF-α, IL-17, IL-22, and IFN-γ, while promoting peripheral immune tolerance via checkpoint ligands PD-L1 and PD-L2 [15].

Recent studies have demonstrated that MSC-EVs can attenuate graft-versus-host disease (GVHD) and support hematopoietic recovery by modulating antigen-presenting cells, regulatory T cells, and cytokine networks [39,49]. These findings reinforce the concept of MSC-derived vesicles as potent, cell-free therapeutic agents bridging regenerative and immunomodulatory medicine.

## 4. Clinical Applications of MSCs After Allo-HSCT

### 4.1. Pathophysiology and Current Therapies

Graft-versus-host disease (GVHD) arises from donor T-cell-mediated immune attack on host tissues after allogeneic HSCT, primarily affecting the skin, liver, and gastrointestinal tract. It is traditionally divided into acute (aGVHD) and chronic (cGVHD) forms, though the distinction is often arbitrary due to overlapping features. GVHD contributes to 8–16% of transplant-related mortality, mainly via infection or organ failure [50,51,52]. Conditioning-induced tissue injury releases pro-inflammatory cytokines that prime donor T cells to recognize host antigens and recruit cytotoxic effectors, resulting in tissue damage. Chronic GVHD is driven by sustained inflammation, T- and B-cell dysregulation, and profibrotic cytokines such as PDGFα and TGFβ, while elevated CXCL9, CXCL10, and BAFF levels correlate with disease severity [53,54,55,56].

Corticosteroids remain first-line therapy, yet outcomes are poor in steroid-refractory cases. Ruxolitinib Janus Kinase (JAK) inhibitor is FDA-approved for SR-aGVHD [57] and SR-cGVHD, whereas belumosudil Rho-associated coiled-coil containing protein kinase 2 (ROCK2) inhibitor is approved for cGVHD [58]. Despite these advances, up to 60% of patients eventually require additional steroid-sparing options [59].

### 4.2. MSCs in Steroid-Refractory aGVHD

Preclinical data demonstrating the potent immunomodulatory properties of MSCs led to their clinical application in SR-aGVHD [11]. The first successful case (2004) reported complete remission of severe gastrointestinal and hepatic GVHD after infusion of haploidentical maternal MSCs [11]. In the pivotal European phase II trial, 55 patients achieved an overall response (OR) of 71%, with complete responders showing improved survival [60]. Meta-analyses confirmed favorable safety, minimal toxicity, and no increase in relapse risk, though isolated late infections occurred [61,62,63,64,65].

Subsequent phase II–III studies, including the U.S. multicenter Remestemcel-L (Prochymal) trial, demonstrated excellent tolerability but failed to meet primary endpoints; subgroup analyses suggested higher responses in hepatic GVHD and in pediatric patients [66,67]. Consistent multicenter data across Europe and Asia reported ORs between 48% and 71% and survival benefit among responders [35,68,69,70,71,72,73]. Real-world evidence from Japan with Temcell showed organ-specific ORs (36–64%) and better outcomes in younger recipients and early-stage disease [74]. Recent phase II/III studies [39,75] reaffirm the excellent safety profile and highlight the predictive value of early response.

Despite these consistent clinical observations, response patterns differ between acute and chronic GVHD, largely reflecting their distinct immunopathology. Acute GVHD is driven by a highly inflammatory cytokine cascade (TNF-α, IFN-γ, IL-6) that “licenses’’ MSCs to exert potent immunosuppressive functions via IDO, PGE_2_, and HLA-G pathways, contributing to higher OR rates in SR-aGVHD [76]. In contrast, chronic GVHD involves aberrant B-cell activation, impaired thymic repair, and progressive fibrosis, creating a less pro-inflammatory microenvironment that may reduce MSC responsiveness and lead to more variable outcomes [77]. Pediatric patients consistently achieve higher response rates, likely due to reduced immune senescence, and fewer comorbidities, which facilitate Treg expansion and enhance MSC-mediated immunoregulation [78,79,80]. These biological differences help contextualize the heterogeneity of MSC efficacy across GVHD subtypes and age groups.

As summarized in Table 1, MSC therapy offers a well-tolerated adjunctive option for SR-aGVHD, although efficacy varies with patient age, disease severity, and infusion timing.

### 4.3. Preemptive and Prophylactic MSC Administration

Preventive or pre-emptive MSC infusion following allo-HSCT has gained attention for mitigating post-transplant immune complications. Multicenter randomized trials demonstrate reduced acute and chronic GVHD incidence, especially in haploidentical settings. Gao et al. reported lower 2-year cGVHD (27.4% vs. 49.0%, *p* = 0.021), supporting early MSC-mediated immune modulation [84]. Wang et al. confirmed that a single pre-graft MSC dose accelerates platelet engraftment and decreases severe aGVHD without increasing relapse risk [85].

More recent RCTs, including Lombardo et al. (2024), demonstrated good safety and a trend toward reduced grade II–IV aGVHD, while Huang et al. (2024) and Yao et al. (2025) showed decreased severe cGVHD and improved graft-versus-host-free, relapse-free survival (GRFS). Although optimal dose, timing, and patient selection criteria remain under clarification, MSC-based prophylaxis represents a promising avenue to improve long-term immune tolerance and survival after allo-HSCT [84,85,86,87,88]. Collectively, as summarized in Table 2, preemptive MSC administration mitigates harmful alloreactivity without impairing graft-versus-leukemia effects.

### 4.4. MSC for the Treatment of Poor Graft Function

Poor graft function (PGF) is an uncommon but clinically significant complication following allogeneic hematopoietic cell transplantation (allo-HSCT). It is defined by persistent cytopenias in the presence of full donor chimerism and absence of relapse, infection, or GVHD [89]. PGF results in recurrent infections, bleeding, transfusion dependence, and increased healthcare utilization, underscoring its substantial morbidity [89,90]. Current therapeutic approaches—including thrombopoietin receptor agonists (TPO-RAs), donor stem cell boosts, and second transplantation—are limited by variable efficacy and potential risks such as GVHD or additional toxicity [91,92,93].

Given the central role of bone marrow stromal integrity in supporting hematopoiesis, interest has grown in mesenchymal stromal/stem cells (MSCs) as a stromal-restorative therapy for PGF. Damage to the marrow microenvironment may result from conditioning intensity, prior therapy, immune-mediated injury, or chronic inflammation [93]. MSCs secrete hematopoietic cytokines and growth factors (e.g., SCF, IL-6, CXCL12), modulate immune responses, and support hematopoietic stem and progenitor cell (HSPC) survival and function, making them a biologically plausible intervention for PGF [94]. Mechanistic observations from Song et al. demonstrated impaired proliferation and niche-supportive capacity in endogenous MSCs from PGF patients, further supporting the rationale for exogenous MSC administration [95]. However, these findings remain exploratory and require validation in larger cohorts.

Clinical investigations of MSCs in PGF have yielded heterogeneous but generally encouraging signals. In a prospective study of 20 PGF patients, Liu et al. reported hematologic improvement in 85% following one to three infusions of bone marrow-derived MSCs, with reduced transfusion needs and increases in ANC and platelets [12]. In contrast, Zhao et al. observed limited hematologic recovery in primary PGF despite MSC therapy, with 1-year overall survival markedly lower than in patients with good graft function (25% vs. 90.6%), highlighting the severe natural history of primary PGF and the need for earlier or combined therapeutic strategies [96]. More recently, Servais et al. reported overall response rates of 53% by day + 90 and 67% by day + 180, along with substantial reductions in transfusion requirements, suggesting that MSCs may provide clinically meaningful benefit in selected patients [75].

As summarized in Table 3, evidence to date indicates that MSC therapy is feasible and generally well tolerated, though response rates vary across studies and patient populations. Importantly, most available data derive from small, non-randomized cohorts, limiting definitive conclusions. Larger controlled trials are needed to clarify optimal dosing, timing, patient selection, and the potential role of combinatorial approaches (e.g., MSCs with TPO-RAs [97]) in improving hematopoietic recovery in PGF.

## 5. Conclusions and Future Perspectives

Mesenchymal stromal/stem cells (MSCs) possess strong immunomodulatory and trophic properties, making them biologically attractive candidates for managing immune complications and stromal injury after allogeneic HSCT. Current clinical data—especially in steroid-refractory aGVHD—indicate that MSC therapy is generally safe and may provide clinically meaningful responses in selected patients. However, study outcomes remain inconsistent, and reproducible benefits across broader patient populations have yet to be demonstrated.

A key challenge is the intrinsic heterogeneity of MSCs. Donor variability, tissue source, culture conditions, passage number, and inflammatory priming significantly influence MSC potency, contributing to the variability seen across trials. Additionally, infused MSCs exhibit limited persistence and suboptimal homing, features heavily shaped by the inflammatory and metabolic conditions of the recipient. These biological constraints underline the need for standardized manufacturing protocols, validated potency assays, and biomarker-guided strategies for dose, timing, and patient selection.

MSC-derived extracellular vesicles (MSC-EVs) have emerged as a promising cell-free alternative with theoretical advantages in safety and scalability. Nevertheless, the clinical development of MSC-EVs remains at an early stage, and robust randomized evidence is required before they can be considered alongside or instead of cellular MSC products.

Moving forward, well-designed prospective studies, harmonized production and quality-control pipelines, and deeper mechanistic insight into MSC–immune interactions will be essential to determine which patients are most likely to benefit. Advances such as preconditioning, genetic enhancement, or biomaterial-supported delivery systems may ultimately improve MSC persistence and functional stability. Until such evidence becomes available, MSC-based therapies should be considered with cautious optimism and applied within controlled or well-defined clinical contexts.

## 6. Key Take-Home Messages

MSC therapy shows promising immunomodulatory and stromal-restorative effects, but clinical responses remain inconsistent due to product heterogeneity, patient variability, and limited in vivo persistence.

MSC-derived extracellular vesicles (EVs) offer a potentially safer, more standardized cell-free alternative, yet require robust clinical validation before widespread adoption.

Future progress depends on standardization—including GMP-compatible manufacturing, potency assays, optimized dosing/timing, and biomarker-based patient selection—to enable reliable, reproducible, and evidence-based MSC use after allo-HSCT.

## Figures and Tables

**Table 1:** Selected clinical trials of MSC therapy in SR-aGVHD.

Study (Year)	Population	MSC Dose and Schedule	Overall Response (OR)	Survival Outcome	Clinical Relevance/Key Message
Le Blanc 2008 [60]	*n* = 55	Med 1.4 × 10^6^ cells/kg; 1–5 doses	70.8%	2-y OS = 35%	Early study showing feasibility and moderate survival.
Introna 2014 [49]	*n* = 40	Med 1.5 × 10^6^ cells/kg; 2–11 doses	60%	2-y OS 50% (children)	Pediatric subgroup benefited most
Fernández-Maqueda 2017 [68]	*n* = 33	Med 1.06 × 10^6^ cells/kg; 1–16 doses	81%	1-y OS 79% in CR patients	Response quality strongly predicts survival
Servais 2018 [81]	*n* = 33	1–4 × 10^6^ cells/kg; 1–2 dose	40.6%	1-y OS = 18%	Salvage option; early MSC timing improves outcomes.
Ke Zhao 2022 [82]	*n* = 101(T) vs. 102(C)	1 × 10^6^ cells/kg; q1w × 4	82.9%	3-y OS = 63.4 vs. 48.5%	MSCs improved OR and FFS and reduced cGVHD without increasing relapse.
Ulu 2025 [83]	*n* = 36	Med 1.72 × 10^6^ cells/kg; q2w × 2	~20% at 1 mo	6-mo OS = 33.3%	Safe; better responses in younger/higher-PLT patients, but limited efficacy.

Abbreviations: cGVHD = chronic graft-versus-host disease; C = control arm; CR = complete response; FFS = failure-free survival; Med = median; MSC = mesenchymal stromal/stem cell; mo = month; OR = overall response; OS = overall survival; q1w = weekly; q2w = every 2 weeks; SR-aGVHD = steroid-refractory acute graft-versus-host disease; T = treatment arm; vs. = versus; y = year.

**Table 2:** Preemptive or prophylactic MSC use after allo-HSCT: selected clinical trials.

Study (Year)	Population	MSC Dose and Schedule	Outcomes	Clinical Relevance/Key Message
Gao 2016 [84]	*n* = 62 (T) vs. 62 (C)	3 × 10^7^ cells/kg; mo × 2–4 doses	2-year cumulative cGvHD 27.4% vs. 49.0% (*p* = 0.021)	First multicenter RCT showing prophylactic benefit on cGvHD after haplo-HSCT.
Wang 2020 [85]	*n* = 25 (T) vs. 25 (C)	1 × 10^6^ cells/kg; × 1 dose	Faster platelet engraftment; ↓ severe aGvHD; no ↑ relapse	Pre-infusion strategy with engraftment and GVHD advantages.
Lombardo 2024 [86]	*n* = 60 (T) vs. 60 (C)	1.5–3 × 10^6^ cells/kg; ×1 dose	Trend toward ↓ grade II–IV aGvHD; good safety	Only double-blind RCT of MSC co-infusion at HSC infusion; signals benefit, safe profile.
Huang 2024 [87]	*n* = 74 (T) vs. 74 (C)	1 × 10^6^ cells/kg; q2w × 4 (~day + 45 & + 100)	↓ severe cGvHD; better GRFS	Early, MSCs infusions reduce severe cGvHD and improve GRFS after haplo-HSCT.
Yao 2025 [88]	*n* = 96 (T) vs. 96 (C)	1 × 10^6^ cells/kg; 8 doses/3 mo	↓ aGvHD & cGvHD; ↑ GRFS	Early sequential MSCs improved GVHD control and GRFS.

Abbreviations: aGvHD = acute graft-versus-host disease; allo-HSCT = allogeneic hematopoietic stem cell transplantation; C = control arm; cGvHD = chronic graft-versus-host disease; GRFS = graft-versus-host-free, relapse-free survival; GVHD = graft-versus-host disease; HSC = hematopoietic stem cell; mo = month; MSC = mesenchymal stromal/stem cell; q2w = biweekly; RCT = randomized controlled trial; T = treatment arm; vs. = versus; ↓ = decreased; ↑ = increased

**Table 3:** Selected Clinical Studies in PGF: Design, Response and Clinical Relevance.

Study (Year)	Population	MSC Dose and Schedule	Hematologic Response/Outcome	Clinical Relevance
Liu 2014 [12]	*n* = 20 PGF	~1 × 10^6^ cells/kg; 1–3 dose at 28-day intervals	Hematologic improvement in 85% of patients; increases in ANC and PLT; reduced transfusion requirements	First prospective pilot study demonstrating MSC-mediated hematopoietic rescue in PGF; acceptable safety
Zhao 2019 [96]	*n* = 24 PGF	1 × 10^6^ cells/kg; wk × 2; third dose if no response)	Limited recovery; persistent cytopenias in primary PGF.	Primary PGF associated with high mortality; highlights unmet need for effective therapies such as MSC
Servais 2023 [75]	*n* = 30 PGF	1–2 × 10^6^ cells/kg; ×1	Major reduction in RBC/PLT transfusion; ANC recovery	Single-dose BM-MSC appears effective and safe for PGF; supports MSC as salvage option

Abbreviations: ANC = absolute neutrophil count; BM-MSC = bone marrow–derived mesenchymal stromal/stem cell; MSC = mesenchymal stromal/stem cell; PGF = poor graft function; PLT = platelet; RBC = red blood cell; wk = week.

## Data Availability

No new data were created or analyzed in this study. Data sharing is not applicable to this article.

## References

[1] Salem H.K., Thiemermann C. (2010). Mesenchymal stromal cells: Current understanding and clinical status. Stem Cells.

[2] Spees J.L., Olson S.D., Ylostalo J., Lynch P.J., Smith J., Perry A., Peister A., Wang M.Y., Prockop D.J. (2003). Differentiation, cell fusion, and nuclear fusion during ex vivo repair of epithelium by human adult stem cells from bone marrow stroma. Proc. Natl. Acad. Sci. USA.

[3] Phinney D.G., Isakova I. (2005). Plasticity and therapeutic potential of mesenchymal stem cells in the nervous system. Curr. Pharm. Des..

[4] Sivamani R.K., Schwartz M.P., Anseth K.S., Isseroff R.R. (2011). Keratinocyte proximity and contact can play a significant role in determining mesenchymal stem cell fate in human tissue. FASEB J..

[5] Reyhani S., Abbaspanah B., Mousavi S.H. (2020). Umbilical cord-derived mesenchymal stem cells in neurodegenerative disorders: From literature to clinical practice. Regen. Med..

[6] Yang G., Fan X., Mazhar M., Yang S., Xu H., Dechsupa N., Wang L. (2022). Mesenchymal stem cell application and its therapeutic mechanisms in intracerebral hemorrhage. Front. Cell. Neurosci..

[7] Leuning D.G., Beijer N.R.M., du Fossé N.A., Vermeulen S., Lievers E., van Kooten C., Rabelink T.J., Boer J. (2018). The cytokine secretion profile of mesenchymal stromal cells is determined by surface structure of the microenvironment. Sci. Rep..

[8] Viswanathan S., Shi Y., Galipeau J., Krampera M., Leblanc K., Martin I., Nolta J., Phinney D.G., Sensebe L. (2019). Mesenchymal stem versus stromal cells: International Society for Cell & Gene Therapy (ISCT^®^) Mesenchymal Stromal Cell Committee position statement on nomenclature. Cytotherapy.

[9] Li N., Hua J. (2017). Interactions between mesenchymal stem cells and the immune system. Cell. Mol. Life Sci..

[10] Uccelli A., Moretta L., Pistoia V. (2008). Mesenchymal stem cells in health and disease. Nat. Rev. Immunol..

[11] Le Blanc K., Rasmusson I., Sundberg B., Götherström C., Hassan M., Uzunel M., Ringdén O. (2004). Treatment of severe acute graft-versus-host disease with third party haploidentical mesenchymal stem cells. Lancet.

[12] Liu X., Wu M., Peng Y., Chen X., Sun J., Huang F., Fan Z., Zhou H., Wu X., Yu G. (2014). Improvement in poor graft function after allogeneic hematopoietic stem cell transplantation upon administration of mesenchymal stem cells from third-party donors: A pilot prospective study. Cell Transplant..

[13] Huang Y., Wu Q., Tam P.K.H. (2022). Immunomodulatory mechanisms of mesenchymal stem cells and their potential clinical applications. Int. J. Mol. Sci..

[14] Davies L.C., Heldring N., Kadri N., Le Blanc K. (2017). Mesenchymal stromal cell secretion of programmed death-1 ligands regulates T cell mediated immunosuppression. Stem Cells.

[15] Tang Y., Zhou Y., Li H.J. (2021). Advances in mesenchymal stem cell exosomes: A review. Stem Cell Res. Ther..

[16] Han X., Liao R., Li X., Zhang C., Huo S., Qin L., Xiong Y., He T., Xiao G., Zhang T. (2025). Mesenchymal stem cells in treating human diseases: Molecular mechanisms and clinical studies. Signal Transduct. Target. Ther..

[17] Caplan A.I. (1991). Mesenchymal stem cells. J. Orthop. Res..

[18] Mahla R.S. (2016). Stem cells applications in regenerative medicine and disease therapeutics. Int. J. Cell Biol..

[19] Galipeau J., Sensébé L. (2018). Mesenchymal stromal cells: Clinical challenges and therapeutic opportunities. Cell Stem Cell.

[20] Sobacchi C., Palagano E., Villa A., Menale C. (2017). Soluble factors on stage to direct mesenchymal stem cells fate. Front. Bioeng. Biotechnol..

[21] Li G., Kitada M., Dezawa M. (2025). Hypoxia Boosts Pluripotent-Like Muse Cell Ratio in Mesenchymal Stromal Cells and Upregulates Pluripotency Gene Expression. Sci. Rep..

[22] Li J., Wu Z., Zhao L., Liu Y., Su Y., Gong X., Liu F., Zhang L. (2023). The Heterogeneity of Mesenchymal Stem Cells: An Important Issue to Be Addressed in Cell Therapy. Stem Cell Res. Ther..

[23] Zhou Y., Yamamoto Y., Xiao Z., Ochiya T. (2019). The immunomodulatory functions of mesenchymal stromal/stem cells mediated via paracrine activity. J. Clin. Med..

[24] Chen J., Zheng C.X., Jin Y., Hu C.H. (2021). Mesenchymal stromal cell-mediated immune regulation: A promising remedy in the therapy of type 2 diabetes mellitus. Stem Cells.

[25] Reis M., Mavin E., Nicholson L., Green K., Dickinson A.M., Wang X.N. (2018). Mesenchymal stromal cell-derived extracellular vesicles attenuate dendritic cell maturation and function. Front. Immunol..

[26] Lu D., Xu Y., Liu Q., Zhang Q. (2021). Mesenchymal stem cell-macrophage crosstalk and maintenance of inflammatory microenvironment homeostasis. Front. Cell Dev. Biol..

[27] Vasandan A.B., Jahnavi S., Shashank C., Prasad P., Kumar A., Prasanna S.J. (2016). Human mesenchymal stem cells program macrophage plasticity by altering their metabolic status via a PGE2-dependent mechanism. Sci. Rep..

[28] Salami F., Tavassoli A., Mehrzad J., Parham A. (2018). Immunomodulatory effects of mesenchymal stem cells on leukocytes with emphasis on neutrophils. Immunobiology.

[29] Guo R., Liang Q., He Y., Wang C., Jiang J., Chen T., Zhang D., Hu K. (2023). Mesenchymal stromal cells-derived extracellular vesicles regulate dendritic cell functions in dry eye disease. Cells.

[30] Gao F., Chiu S.M., Motan D.A., Zhang Z., Chen L., Ji H.L., Tse H.F., Fu Q.L., Lian Q. (2016). Mesenchymal stem cells and immunomodulation: Current status and future prospects. Cell Death Dis..

[31] Jiang W., Xu J. (2020). Immune modulation by mesenchymal stem cells. Cell Prolif..

[32] Sakaguchi S., Yamaguchi T., Nomura T., Ono M. (2008). Regulatory T cells and immune tolerance. Cell.

[33] Di Ianni M., Del Papa B., De Ioanni M., Moretti L., Bonifacio E., Cecchini D., Sportoletti P., Falzetti F., Tabilio A. (2008). Mesenchymal cells recruit and regulate T regulatory cells. Exp. Hematol..

[34] Del Papa B., Sportoletti P., Cecchini D., Rosati E., Balucani C., Baldoni S., Fettucciari K., Marconi P., Martelli M.F., Falzetti F. (2013). Notch1 modulates mesenchymal stem cells mediated regulatory T-cell induction. Eur. J. Immunol..

[35] Hong J.W., Lim J.H., Chung C.J., Kang T.J., Kim T.Y., Kim Y.S., Roh T.S., Lew D.H. (2017). Immune tolerance of human dental pulp-derived mesenchymal stem cells mediated by CD4⁺CD25⁺FoxP3⁺ regulatory T-cells and induced by TGF-β1 and IL-10. Yonsei Med. J..

[36] Kadri N., Amu S., Iacobaeus E., Boberg E., Le Blanc K. (2023). Current perspectives on mesenchymal stromal cell therapy for graft versus host disease. Cell. Mol. Immunol..

[37] Gieseke F., Böhringer J., Bussolari R., Dominici M., Handgretinger R., Müller I. (2010). Human multipotent mesenchymal stromal cells use galectin-1 to inhibit immune effector cells. Blood.

[38] Laranjeira P., Pedrosa M., Pedreiro S., Gomes J., Martinho A., Antunes B., Ribeiro T., Santos F., Trindade H., Paiva A. (2015). Effect of human bone marrow mesenchymal stromal cells on cytokine production by peripheral blood naive, memory, and effector T cells. Stem Cell Res. Ther..

[39] Draguet F., Bouland C., Dubois N., Bron D., Meuleman N., Stamatopoulos B., Lagneaux L. (2023). Potential of mesenchymal stromal cell-derived extracellular vesicles as natural nanocarriers: Concise review. Pharmaceutics.

[40] Liotta F., Angeli R., Cosmi L., Filì L., Manuelli C., Frosali F., Mazzinghi B., Maggi L., Pasini A., Lisi V. (2008). Toll-like receptors 3 and 4 are expressed by human bone marrow-derived mesenchymal stem cells and can inhibit their T-cell modulatory activity by impairing Notch signaling. Stem Cells.

[41] Spees J.L., Lee R.H., Gregory C.A. (2016). Mechanisms of mesenchymal stem/stromal cell function. Stem Cell Res. Ther..

[42] Wu Y., Chen L., Scott P.G., Tredget E.E. (2007). Mesenchymal stem cells enhance wound healing through differentiation and angiogenesis. Stem Cells.

[43] Lai R.C., Arslan F., Lee M.M., Sze N.S., Choo A., Chen T.S., Salto-Tellez M., Timmers L., Lee C.N., El Oakley R.M. (2010). Exosome secreted by MSC reduces myocardial ischemia/reperfusion injury. Stem Cell Res..

[44] Rani S., Ryan A.E., Griffin M.D., Ritter T. (2015). Mesenchymal stem cell-derived extracellular vesicles: Toward cell-free therapeutic applications. Mol. Ther..

[45] Raposo G., Stoorvogel W. (2013). Extracellular vesicles: Exosomes, microvesicles, and friends. J. Cell Biol..

[46] Raposo G., Nijman H.W., Stoorvogel W., Liejendekker R., Harding C.V., Melief C.J., Geuze H.J. (1996). B lymphocytes secrete antigen-presenting vesicles. J. Exp. Med..

[47] Skog J., Würdinger T., van Rijn S., Meijer D.H., Gainche L., Sena-Esteves M., Curry W.T., Carter B.S., Krichevsky A.M., Breakefield X.O. (2008). Glioblastoma microvesicles transport RNA and proteins that promote tumour growth and provide diagnostic biomarkers. Nat. Cell Biol..

[48] Clancy J.W., Schmidtmann M., D’Souza-Schorey C. (2021). The ins and outs of microvesicles. FASEB Bioadv..

[49] Introna M., Lucchini G., Dander E., Galimberti S., Rovelli A., Balduzzi A., Longoni D., Pavan F., Masciocchi F., Algarotti A. (2014). Treatment of graft versus host disease with mesenchymal stromal cells: A phase I study on 40 adult and pediatric patients. Biol. Blood Marrow Transplant..

[50] Garnett C., Apperley J.F., Pavlů J. (2013). Treatment and management of graft-versus-host disease: Improving response and survival. Ther. Adv. Hematol..

[51] Socié G., Ritz J. (2014). Current issues in chronic graft-versus-host disease. Blood.

[52] Phelan R., Arora M., Chen M. (2020). Current Use and Outcome of Hematopoietic Stem Cell Transplantation: CIBMTR US Summary Slides. https://www.cibmtr.org/ReferenceCenter/SlidesReports/SummarySlides/Pages/index.aspx.

[53] Wolff D., Greinix H., Lee S.J., Gooley T., Paczesny S., Pavletic S., Hakim F., Malard F., Jagasia M., Lawitschka A. (2018). Biomarkers in chronic graft-versus-host disease: Quo vadis?. Bone Marrow Transplant..

[54] Goklemez S., Im A.P., Cao L., Pirsl F., Steinberg S.M., Curtis L.M., Mitchell S.A., Cowen E.W., Baruffaldi J., Rose J. (2020). Clinical characteristics and cytokine biomarkers in patients with chronic graft-vs-host disease persisting seven or more years after diagnosis. Am. J. Hematol..

[55] Subburaj D., Ng B., Kariminia A., Abdossamadi S., Lauener M., Nemecek E.R., Rozmus J., Kharbanda S., Kitko C.L., Lewis V.A. (2022). Metabolomic identification of α-ketoglutaric acid elevation in pediatric chronic graft-versus-host disease. Blood.

[56] Cuvelier G.D.E., Ng B., Abdossamadi S., Nemecek E.R., Melton A., Kitko C.L., Lewis V.A., Schechter T., Jacobsohn D.A., Harris A.C. (2023). A diagnostic classifier for pediatric chronic graft-versus-host disease: Results of the ABLE/PBMTC 1202 study. Blood Adv..

[57] Food and Drug Administration (2019). FDA Approves Ruxolitinib for Acute Graft Versus-Host Disease. https://www.fda.gov/drugs/resources-information-approved-drugs/fda-approves-ruxolitinib-acute-graft-versus-host-disease.

[58] Food and Drug Administration (2021). FDA Approves Ruxolitinib for Chronic Graft-Versus-Host Disease. https://www.fda.gov/drugs/resources-information-approved-drugs/fda-approves-ruxolitinib-chronic-graft-versus-host-disease.

[59] Flowers M.E., Martin P.J. (2015). How we treat chronic graft-versus-host disease. Blood.

[60] Le Blanc K., Frassoni F., Ball L., Locatelli F., Roelofs H., Lewis I., Lanino E., Sundberg B., Bernardo M.E., Remberger M. (2008). Mesenchymal stem cells for treatment of steroid-resistant, severe, acute graft-versus-host disease: A phase II study. Lancet.

[61] Bader P., Kuçi Z., Bakhtiar S., Basu O., Bug G., Dennis M., Greil J., Barta A., Kállay K.M., Lang P. (2018). Effective treatment of steroid and therapy-refractory acute graft-versus-host disease with a novel mesenchymal stromal cell product (MSC-FFM). Bone Marrow Transplant..

[62] Lalu M.M., McIntyre L., Pugliese C., Fergusson D., Winston B.W., Marshall J.C., Granton J., Stewart D.J., Canadian Critical Care Trials Group (2012). Safety of cell therapy with mesenchymal stromal cells (SafeCell): A systematic review and meta-analysis of clinical trials. PLoS ONE.

[63] Müller I., Kordowich S., Holzwarth C., Isensee G., Lang P., Neunhoeffer F., Dominici M., Greil J., Handgretinger R. (2008). Application of multipotent mesenchymal stromal cells in pediatric patients following allogeneic stem cell transplantation. Blood Cells Mol. Dis..

[64] von Bahr L., Batsis I., Moll G., Hägg M., Szakos A., Sundberg B., Uzunel M., Ringden O., Le Blanc K. (2012). Analysis of tissues following mesenchymal stromal cell therapy in humans indicates limited long-term engraftment and no ectopic tissue formation. Stem Cells.

[65] Stoma I., Karpov I., Krivenko S., Iskrov I., Milanovich N., Koritko A., Uss A. (2018). Mesenchymal stem cells transplantation in hematological patients with acute graft-versus-host disease: Characteristics and risk factors for infectious complications. Ann. Hematol..

[66] Kebriaei P., Hayes J., Daly A., Uberti J., Marks D.I., Soiffer R., Waller E.K., Burke E., Skerrett D., Shpall E. (2020). A phase 3 randomized study of Remestemcel-L versus placebo added to second-line therapy in patients with steroid-refractory acute graft-versus-host disease. Biol. Blood Marrow Transplant..

[67] Ringdén O., Uzunel M., Rasmusson I., Remberger M., Sundberg B., Lönnies H., Marschall H.U., Dlugosz A., Szakos A., Hassan Z. (2006). Mesenchymal stem cells for treatment of therapy-resistant graft-versus-host disease. Transplantation.

[68] Fernández-Maqueda C., Gonzalo-Daganzo R., Regidor C., Martín-Donaire T., Sánchez R., Bueno J.L., Bautista G., De Liglesia A., Gutiérrez Y., García-Berciano M. (2017). Mesenchymal stromal cells for steroid-refractory acute GvHD. Bone Marrow Transplant..

[69] Muroi K., Miyamura K., Okada M., Yamashita T., Murata M., Ishikawa T., Uike N., Hidaka M., Kobayashi R., Imamura M. (2016). Bone marrow-derived mesenchymal stem cells (JR-031) for steroid-refractory grade III or IV acute graft-versus-host disease: A phase II/III study. Int. J. Hematol..

[70] Zhao K., Lou R., Huang F., Peng Y., Jiang Z., Huang K., Wu X., Zhang Y., Fan Z., Zhou H. (2015). Immunomodulation effects of mesenchymal stromal cells on acute graft-versus-host disease after hematopoietic stem cell transplantation. Biol. Blood Marrow Transplant..

[71] Salmenniemi U., Itälä-Remes M., Nystedt J., Putkonen M., Niittyvuopio R., Vettenranta K., Korhonen M. (2017). Good responses but high TRM in adult patients after MSC therapy for GvHD. Bone Marrow Transplant..

[72] Galleu A., Milojkovic D., Deplano S., Szydlo R., Loaiza S., Wynn R., Marks D.I., Richardson D., Orchard K., Kanfer E. (2019). Mesenchymal stromal cells for acute graft-versus-host disease: Response at 1 week predicts probability of survival. Br. J. Haematol..

[73] Sánchez-Guijo F., Caballero-Velázquez T., López-Villar O., Redondo A., Parody R., Martínez C., Olavarría E., Andreu E., Prósper F., Díez-Campelo M. (2014). Sequential third-party mesenchymal stromal cell therapy for refractory acute graft-versus-host disease. Biol. Blood Marrow Transplant..

[74] Murata M., Terakura S., Wake A., Miyao K., Ikegame K., Uchida N., Kataoka K., Miyamoto T., Onizuka M., Eto T. (2021). Off-the-shelf bone marrow-derived mesenchymal stem cell treatment for acute graft-versus-host disease: Real-world evidence. Bone Marrow Transplant..

[75] Servais S., Baron F., Lechanteur C., Seidel L., Baudoux E., Briquet A., Selleslag D., Maertens J., Poire X., Schroyens W. (2023). Multipotent mesenchymal stromal cells as treatment for poor graft function after allogeneic hematopoietic cell transplantation: A multicenter prospective analysis. Front. Immunol..

[76] Zeiser R., Blazar B.R. (2017). Acute graft-versus-host disease—Biologic process, prevention, and therapy. N. Engl. J. Med..

[77] Wolff D., Fatobene G., Rocha V., Kröger N., Flowers M.E. (2021). Steroid-refractory chronic graft-versus-host disease: Treatment options and patient management. Bone Marrow Transplant..

[78] Weinberger B. (2017). Immunosenescence: The importance of considering age in health and disease. Clin. Exp. Immunol..

[79] Kurtzberg J., Prockop S., Teira P., Bittencourt H., Lewis V., Chan K.W., Horn B., Yu L., Talano J.A., Nemecek E. (2014). Allogeneic human mesenchymal stem cell therapy (remestemcel-L, Prochymal) as a rescue agent for severe refractory acute graft-versus-host disease in pediatric patients. Biol. Blood Marrow Transplant..

[80] von Dalowski F., Kramer M., Wermke M., Wehner R., Röllig C., Alakel N., Stölzel F., Parmentier S., Sockel K., Krech M. (2016). Mesenchymal stromal cells for treatment of acute steroid-refractory graft versus host disease: Clinical responses and long-term outcome. Stem Cells.

[81] Servais S., Baron F., Lechanteur C., Seidel L., Selleslag D., Maertens J., Baudoux E., Zachee P., Van Gelder M., Noens L. (2018). Infusion of bone marrow derived multipotent mesenchymal stromal cells for the treatment of steroid-refractory acute graft-versus-host disease: A multicenter prospective study. Oncotarget.

[82] Zhao K., Lin R., Fan Z., Chen X., Wang Y., Huang F., Xu N., Zhang X., Zhang X., Xuan L. (2022). Mesenchymal stromal cells plus basiliximab, calcineurin inhibitor as treatment of steroid-resistant acute graft-versus-host disease: A multicenter, randomized, phase 3, open-label trial. J. Hematol. Oncol..

[83] Ulu B.U., Hindilerden I.Y., Yigenoglu T.N., Tiryaki T.O., Erkurt M.A., Korkmaz G., Namdaroglu S., Aksoy E., Korkmaz S., Seyhan M. (2025). Are mesenchymal stem cells still effective in acute GvHD management?. Transfus. Apher. Sci..

[84] Gao L., Zhang Y., Hu B., Liu J., Kong P., Lou S., Su Y., Yang T., Li H., Liu Y. (2016). Phase II multicenter, randomized, double-blind controlled study of efficacy and safety of umbilical cord-derived mesenchymal stromal cells in the prophylaxis of chronic graft-versus-host disease after HLA-haploidentical stem-cell transplantation. J. Clin. Oncol..

[85] Wang X., Zhang M., He P. (2020). Pre-infusion single-dose mesenchymal stem cells promote platelet engraftment and decrease severe acute graft versus host disease without relapse in haploidentical peripheral blood stem cell transplantation. J. Int. Med. Res..

[86] Lombardo G., Lechanteur C., Briquet A., Seidel L., Willems E., Servais S., Baudoux E., Kerre T., Zachee P., Herman J. (2024). Co-infusion of mesenchymal stromal cells to prevent GVHD after allogeneic hematopoietic cell transplantation from HLA-mismatched unrelated donors after reduced-intensity conditioning: A double-blind randomized study and literature review. Stem Cell Res. Ther..

[87] Huang R., Chen T., Wang S., Wang J., Su Y., Liu J., Zhang Y., Ma X., Wen Q., Kong P. (2024). Mesenchymal stem cells for prophylaxis of chronic graft-vs-host disease after haploidentical hematopoietic stem cell transplant: An open-label randomized clinical trial. JAMA Oncol..

[88] Yao H., Huang R., Fu H., Lin R., Zhang Y., Feng Y., Wang Y., Chen T., Wang X., Zhu L. (2025). Sequential infusion of mesenchymal stem cell for graft-versus-host disease prevention in haploidentical hematopoietic stem cell transplantation: An open-label, multicenter, randomized controlled clinical trial. J. Clin. Oncol..

[89] Prabahran A., Koldej R., Chee L., Ritchie D. (2022). Clinical features, pathophysiology, and therapy of poor graft function post-allogeneic stem cell transplantation. Blood Adv..

[90] Prabahran A., Koldej R., Chee L., Wong E., Ritchie D. (2021). Evaluation of risk factors for and subsequent mortality from poor graft function (PGF) post allogeneic stem cell transplantation. Leuk. Lymphoma.

[91] Mahat U., Rotz S.J., Hanna R. (2020). Use of thrombopoietin receptor agonists in prolonged thrombocytopenia after hematopoietic stem cell transplantation. Biol. Blood Marrow Transplant..

[92] Shahzad M., Siddiqui R.S., Anwar I., Chaudhary S.G., Ali T., Naseem M., Ahmed T.F., Ahmed Z., Khurana S., Ahmed N. (2021). Outcomes with CD34-selected stem cell boost for poor graft function after allogeneic hematopoietic stem cell transplantation: A systematic review and meta-analysis. Transplant. Cell Ther..

[93] Man Y., Lu Z., Yao X., Gong Y., Yang T., Wang Y. (2022). Recent advancements in poor graft function following hematopoietic stem cell transplantation. Front. Immunol..

[94] Li T., Luo C., Zhang J., Wei L., Sun W., Xie Q., Liu Y., Zhao Y., Xu S., Wang L. (2021). Efficacy and safety of mesenchymal stem cells co-infusion in allogeneic hematopoietic stem cell transplantation: A systematic review and meta-analysis. Stem Cell Res. Ther..

[95] Song Y., Zhao H.Y., Lyu Z.S., Cao X.N., Shi M.M., Wen Q., Tang F.F., Wang Y., Xu L.P., Zhang X.H. (2018). Dysfunctional bone marrow mesenchymal stem cells in patients with poor graft function after allogeneic hematopoietic stem cell transplantation. Biol. Blood Marrow Transplant..

[96] Zhao Y., Gao F., Shi J., Luo Y., Tan Y., Lai X., Yu J., Huang H. (2019). Incidence, risk factors, and outcomes of primary poor graft function after allogeneic hematopoietic stem cell transplantation. Biol. Blood Marrow Transplant..

[97] Zhu L., Liu J., Kong P., Gao S., Wang L., Liu H., Zhang C., Gao L., Feng Y., Chen T. (2022). Analysis of the efficacy and safety of avatrombopag combined with MSCs for the treatment of thrombocytopenia after allogeneic hematopoietic stem cell transplantation. Front. Immunol..

